# Evaluation of central, steady, maintained fixation grading for predicting inter-eye visual acuity difference to diagnose and treat amblyopia in strabismic patients

**DOI:** 10.4103/0301-4738.53052

**Published:** 2009

**Authors:** Mihir Kothari, Amar Bhaskare, Deepali Mete, Svetlana Toshniwal, Priti Doshi, Shalini Kaul

**Affiliations:** Aditya Jyot Eye Hospital Wadala, Mumbai and Jyotirmay Eye Clinic and Pediatric Low Vision Center, Thane, Maharashtra, India; 1 Mahatme Eye Hospital and Eye Bank, Nagpur, India; 2 Aditya Jyot Eye Hospital Wadala, Mumbai, India

**Keywords:** Amblyopia, fixation preference testing, strabismus

## Abstract

**Background:**

Diagnosis of amblyopia in preverbal strabismic patients is frequently made by binocular fixation preference (BFP) testing. The reports on reliability of BFP are equivocal. This study evaluated the reliability of BFP testing in patients with horizontal strabismus.

**Materials and Methods:**

This prospective observational study included patients with manifest, horizontal, comitant deviation >10 prism diopter (PD). Inter-eye acuity difference (IEAD) was calculated by converting Snellen visual acuity to logMAR and was compared with BFP testing. The fixation behavior of the non-preferred eye was evaluated by a single investigator as central or uncentral, steady or unsteady and maintained or unmaintained. Amblyopia was defined as the IEAD of >0.2 logMAR.

**Results:**

Of total 61 patients 36 were females and 36 had convergent squint, mean age 9.8 years. The correlation of BFP testing with IEAD was good for esotropia and exotropia. The sensitivity, specificity, positive and negative predictive value of central, steady, maintained (CSM) grading was 93%, 78%, 79%, and 93% respectively. Sensitivity and negative predictive values were higher in children aged four to nine years and anisometropia >1 diopter. The correlation between IEAD and lower grades of BFP testing was poor.

**Conclusions:**

CSM grading for BFP testing is useful for the detection of strabismic amblyopia but not useful to differentiate the depth of the amblyopia.

The diagnosis of amblyopia in preverbal strabismic patients is most frequently made by assessment of binocular fixation preference (BFP).[1] Knapp and Moore described fixation testing as early as 1962.[2] Many investigators have found BFP testing reliable to detect inter-eye visual acuity difference (IEAD) to begin amblyopia therapy in strabismic patients.[3–6] Hakim[7] and Atilla *et al.*,[8] reported BFP to be an insensitive, nonspecific and unreliable test to detect IEAD. They wrote that amblyopia treatment should not be initiated solely on the basis of BFP testing. Treatment of strabismic amblyopia on the basis that the sound eye will show strong fixation preference can be hazardous. Fixation preference could be a severe form of eye dominance, and better methods for testing visual acuity in preverbal children are required. Wright *et al.*,[9] found that the findings of Hakim and Atilla *et al.*, are true for deviations <10 prism diopter (PD) and recommended to perform 10 PD base down fixation test for small angle deviations. Nevertheless, Wright found that even 10 PD base down test was unreliable in children >five years as they were aware of second image and alternated their fixation despite the second image being blurred due to amblyopia. Recently, Friedman *et al.,*[10] and Cotter *et al.*,[11] did not find concordance between BFP testing and IEAD in preverbal children with and without the strabismus in population-based studies.

The other methods for assessment of visual acuity in preverbal children include optokinetic nystagmus (OKN), forced preferential looking (FPL) tests and visual evoked potential (VEP). Although the OKN is a short and simple procedure it requires a child's attention and suffers from lack of standardization of speed of the rotating drum and poor correlation with Snellen-type visual acuity.[12–14] Absence of OKN is found to be of unknown significance as some apparently normal infants have absent OKN.[1] Moreover, positive OKN response has been elicited from patients with no visual cortex.[15] The major difficulty with FPL test is false high acuities in patients with both anisometropic[16] and strabismic amblyopia.[17,18] The possible reason for this could be that patients with amblyopia typically have better near visual acuity. Teller Acuity Card test measures grating acuity rather than resolution acuity and there is lack of crowding phenomenon in FPL tests. The third test, VEP is a summed cortical response that results from temporal change in the intensity of the visual stimulus entering the eye. Widespread clinical application is limited as testing equipment is expensive and training is needed to record and interpret responses. Also, VEP has been recorded in patients with absence of the occipital cortex[19] and cortical blindness.[20] This may be due to the contribution of the secondary visual cortices. The exact origin of the waveforms generated in VEP is not clear.[1]

Till recently, 93% pediatric ophthalmologists relied on BFP as the mainstay of detection and treatment of strabismic amblyopia.[1] Hence, this study was undertaken to evaluate the reliability of BFP testing in patients with horizontal strabismus and the impact of the direction of strabismus, age of the patient and anisometropia on the BFP testing.

## Materials and Methods

This prospective masked observational cohort study was performed in the department of pediatric ophthalmology and strabismus of a tertiary teaching eye hospital namely Aditya Jyot Eye Hospital, Maharashtra between March 31, 2007 and January 15, 2008. Patients with manifest, horizontal, comitant ocular deviation more than 10 PD were included. Patients with best-corrected visual acuity of <20/30 in the better eye, structural ocular co-morbidity, latent squint, manifest or latent nystagmus and previously operated eyes were excluded from the study.

An optometrist recorded the best-corrected distance visual acuity on a 20-feet Snellen's chart. A masked, fellowship-trained, pediatric ophthalmologist then performed a detailed ophthalmic examination including the evaluation of fixation behavior.

The fixation behavior was evaluated by having the patient fixate on an accommodative target held at 40cm and with best correction in place. The fixing eye was occluded to force fixation by the squinting (non-preferred) eye. The occlusion was removed after non-preferred eye took up the fixation. If the non-preferred eye continued to fixate after removal of the occlusion and maintained it through the blink on two consecutive attempts, the fixation was graded as central, steady and maintained (CSM). If the eye failed to maintain fixation through a blink or spontaneously changed the fixation to the preferred eye upon removal of the occluder, it was graded as central, steady, unmaintained (CSUM). If covering the preferred eye resulted in the non-preferred eye fixating centrally but the fixation was unsteady i.e. eye wandering off repeatedly despite maintaining occlusion on the preferred eye, the fixation was called unsteady and graded as central, unsteady, unmaintained (CUSUM). If the non-preferred eye did not take up a fixation at all upon occluding the preferred eye it was graded as uncentral, unsteady and unmaintained (UCUSUM) or wandering fixation. Amblyopia was defined as the inter-eye acuity difference of >0.2 logMAR.

Snellen acuity was converted to the logMAR value using Table 1.[21–23] Range of visual acuity, median visual acuity, mean visual acuity and standard deviation for each grade of fixation were calculated. Sensitivity, specificity and predictive values for the diagnosis of amblyopia using CSM grading were calculated from the Bayesian tables. Two-tailed Student's *t*-test was used to determine the statistical significance. Subgroup analysis was not possible due to small sample size.

**Table 1:** LogMAR equivalent of Snellen's visual acuity[20–23]

Snellen's acuity	LogMAR value
20/17	−0.1
20/20	0
20/30	0.2
20/40	0.3
20/60	0.5
20/80	0.6
20/120	0.8
20/200	1.0
16.7/200	1.1
13.3/200	1.2
10/200	1.3
6.7/200	1.5
3.3/200	1.7
Hand movements perception (2.5/200)	1.9
Perception of light only (1.3/200)	2.2

## Results

Of a total of 61 patients, 36 were females and 36 had convergent squint. The mean age of the patients was 9.8 years ± 0.7 (range 4-16 years).

Of a total of 27 patients with CSM fixation, 16 were freely alternating squint with equal dominance in both eyes. Eleven patients had monocular squint with non-dominant eye having CSM fixation.

There was a good correlation of BFP testing with IEAD [Table 2, with statistical power of the study >80%][24] Patients with CSM fixation grade had better vision than that with CSUM than with CUSUM who in turn had vision better than patients with UCUSUM fixation grade. The correlation of the fixation grade and IEAD for both, esotropia and exotropia was good. The IEAD for the fixation grade of CSM and CSUM was not different in esotropia and exotropia [Table 3]. Due to the small number of patients in CUSUM and UCUSUM fixation grades and an associated large standard deviation, a meaningful correlation analysis of these fixation grades with the visual acuity was not done.

**Table 2:** Correlation of fixation grade and inter-eye acuity difference

Fixation Grade (n = 61)	IEAD in LogMAR median/mean ± SD (range)	*P* Value (difference from the immediate next level of fixation grade)
CSM (n = 27)	0/0.3 ± 0.1 3, (0-0.6)	<0.001
CSUM (n = 19)	0.3/0.3 ± 0.4, (0-1.7)	0.1
CUSUM (n = 4)	0.95/0.4 ± 0.05, (0.5-1.5)	0.2
UCUSUM (n = 11)	1.4/0.71 ± 0.48, (0.5-1.5)	-

CSM: Central, steady, maintained, CSUM: Central, steady, unmaintained, CUSUM: Central, unsteady, unmaintained, UCUAUM: Uncentral, unsteady, unmaintained, SD: Standard deviation

**Table 3:** Comparison of correlation of fixation grade and IEAD among esodeviation and exodeviation

Fixation grade	Esodeviation IEAD in LogMAR median/mean ± SD (range)	Exodeviation IEAD in LogMAR median/mean ± SD (range)	*P* value (esodeviation and exodeviation)
CSM	0/0.2 ± 0.1 7, (0-0.6) n = 12	0/0.1 ± 0.1, (0-0.3) n = 15	0.43
CSUM	0.4/0.2 ± 0.31, (0-1.1) n = 13	0.3/0.6 ± 0.6, (0-1.7) n = 16	0.7

CSM: Central, steady, maintained. CSUM: Central, steady, unmaintained, IEAD: Inter-eye acuity difference, SD: Standard deviation

The difference in IEAD between CSM fixation and CSUM was statistically significant (*P* < 0.001). The distribution of true positives and negatives and test positives and negatives is mentioned in Table 4.

**Table 4:** Bayesian table showing distribution of true positives, test positives, true negatives and test negatives

	No amblyopia	Amblyopic
Overall results (N = 61)		
CSM	25	2
No CSM	7	27
Esodeviation (N = 36)		
CSM	11	1
No CSM	5	19
Ecodeviation (N = 25)		
CSM	14	1
No CSM	2	8
Age 4-9 years (N = 28)		
CSM	10	3
No CSM	4	10
Age 10-1 6 years (N = 33)		
CSM	14	0
No CSM	3	17
Anisometropia ≤ 1D (N = 33)		
CSM	12	1
No CSM	3	20
Anisometropia > 1D (N = 33)		
CSM	9	0
No CSM	4	7

CSM: Central, steady, maintained

The sensitivity, specificity, positive predictive value and negative predictive value of CSM grading were excellent [Table 5]. Sensitivity and negative predictive values were higher with children aged four to nine years and patients with anisometropia > 1 diopter (D).

**Table 5:** Performance of fixation grading

	Overall (n = 61) %	Eso (n = 36) %	Exo (n = 25) %	4-9 years (n = 28) %	10-16 years (n = 33) %	Anisometropia ≤1D (n = 41)%	Anisometropia >1D (n = 20)%
Sensitivity	93	95	89	100	85	95	100
Specificity	78	69	88	78	77	85	69
Positive predictive value	79	79	80	71	85	87	64
Negative predictive value	93	92	93	100	77	94	100
Prevalence	47	55	36	35	60	51	35

Eso: Esodeviation, Exo: Exodeviation, D: Diopter

## Discussion

In this study we found that the fixation grade was positively correlated with the IEAD measured in logMAR. The correlation was good for both esotropia and exotropia. The difference between maintained and unmaintained fixation was statistically significant (*P* < 0.001). Though mean IEAD was higher with UCUSUM fixation than CUSUM which in turn was higher than CSUM, the range of IEAD was very large for each fixation grade. Hence, the most important assessment appears to be detection of a maintained fixation to rule out amblyopia. BFP appears to be more of a qualitative test than a quantitative test i.e. CSM grading can reliably detect the presence of amblyopia but may not differentiate a severe amblyopia from a mild or moderate amblyopia. The vision in a patient with fixation grade of UCUSUM ranged from 20/40 to 3/200. In patients with CUSUM grade the vision ranged from 20/30 to hand movement perception and that with CSUM grade ranged from 20/20 to 3/200. Such wide variation in the vision indicates a poor correlation of the fixation grade with the visual acuity. In this situation a clinician cannot rely on the fixation behavior testing (CSM grading) to diagnose the depth of amblyopia. It may be difficult to comment whether for an individual patient improvement in vision after patching therapy would be associated with improvement in fixation grade from UCUSUM to CUSUM to CSUM and finally to CSM. A prospective study would be ideal to assess whether binocular fixation preference from the poorer grade of fixation improves to a better grade with an associated improvement in the visual acuity or lack thereof. However, there has been no study till date to demonstrate this and going by the data from this study, interpreting the depth of amblyopia from the fixation grade in the presence of such poor correlation can be hazardous. Hence, whenever possible BFP testing should be combined with other tests such as Teller Acuity Test or Cardiff Cards or Lea Gratings for the diagnosis of the depth of amblyopia and monitoring the visual acuity in strabismic children.

The negative predictive value and specificity (ability of a test to detect the true negatives) of maintained fixation was high (93% and 78%). This also means, if the deviating eye maintains fixation on BFP testing (test negative), the likelihood that the patient would not be amblyopic is 93%. The predictive value of the positive test was also good (79%). However, a positive predictive value of 79.4% also means that 20.6% patients would be falsely diagnosed to have amblyopia (false-positive rate). A clinician should be aware of this fact and should not unnecessarily delay the surgical treatment of squint.

Two patients with amblyopia had central, steady and maintained fixation (false-negative). One was a 16-year-old boy with 30 PD alternate exotropia and IEAD of 0.3 logMAR and the other was a 12-year-old girl with 20 PD left eye esotropia with IEAD of 0.6 logMAR. There was no significant refractive error or anisometropia. We believe that in the first patient the cause of alternation despite amblyopia could be a small IEAD and in the second case it could be due to a relatively small angle of deviation. In our study, we could not get a reliable history of patching or penalization therapy for amblyopia, which could affect the fixation pattern causing higher false-negatives.

The IEAD for CSM and CSUM grade was not significantly different on BFP testing for esotropia and exotropia. The sensitivity and negative predictive value of BFP testing was higher in children aged four to nine years compared to children aged 10-16 years. These findings were similar to that reported by Campos and Gulli.[25] In their study, out of 57 amblyopes only 29 were able to alternate after attaining equal visual acuity. The non-alternating squints with CSUM fixation grade were found to have abnormalities in VEP. They concluded that there was no relationship between equal visual acuity and alternate fixation in patients who began treatment when they were >three years old. The relationship between alternate fixation and visual acuity is lost later in life. They attributed lack of alternation despite good visual acuity to be the result of cortical competition. More cortical cells are connected to the originally normal eye even after successful treatment of the amblyopic eye in the non-alternating patients.

The BFP testing was better in patients with anisometropia. Anisometropia with higher ametropia in strabismic eye may be associated with denser amblyopia and stronger fixation preference in the better eye. The exceptions are rare and seldom reported in literature.[26]

The fixation grading used by us was different than previous investigators as we omitted the subclasses in CSUM grade. We graded the patients who could not maintain their fixation through the blink into one category. Wright *et al.*[6] and Sener *et al.*,[5] subclassified CSUM fixation grade based on the degree of *unmaintainedness* as 1) holds with difficulty (fixates with the non-preferred eye momentarily but loses fixation instantly), 2) holds fixation briefly (holds fixation briefly for 2 to 3 sec after removal of the cover from the sound eye) and 3) holds fixation well (holds fixation almost all throughout the blink time before refixation to the dominant eye). We found this grading to be too subjective and spurious. Sener *et al*.,[5] have reported that the difference between the two grades, holds fixation with difficulty and holds fixation briefly was not statistically significant.

The limitations of this study include all the shortcomings of a case-control study, a small sample size and lack of data on interobserver and intraobserver variation.

We conclude from this study that CSM grading for BFP testing is useful for the detection of strabismic amblyopia. However, the clinician should also be reminded that if absence of maintained fixation is used as the sole criterion for the diagnosis of amblyopia then nearly 20% patients would be wrongly labeled as having amblyopia thereby causing an unnecessary delay in the surgical treatment and increase in the risk of developing occlusion-induced amblyopia in the fixating eye.[8]
